# HOLOTWIN: A Modular and Interoperable Approach to Holographic Telepresence System Development

**DOI:** 10.3390/s23218692

**Published:** 2023-10-24

**Authors:** Ivaylo Bozhilov, Radostina Petkova, Krasimir Tonchev, Agata Manolova, Vladimir Poulkov

**Affiliations:** Faculty of Telecommunications, Technical University of Sofia, 8 Kliment Ohridski Blvd., 1000 Sofia, Bulgaria; rapetkova@tu-sofia.bg (R.P.); k_tonchev@tu-sofia.bg (K.T.); amanolova@tu-sofia.bg (A.M.); vkp@tu-sofia.bg (V.P.)

**Keywords:** holographic telepresence systems, holographic communication, communication architectures, image processing, virtual reality

## Abstract

In the field of holographic-type communication (HTC), this paper presents a comprehensive exploration of existing technologies and proposes a novel, modular architecture for holographic telepresence systems (HTPSs). We substantiate our architectural framework through a practical implementation, demonstrating its modularity, interoperability, and versatility. Quantitative and qualitative assessments reveal both the promise and areas for improvement within our platform. Our findings reinforce the premise that the key to unlocking HTC’s future lies in modularity and interoperability, serving as critical pillars for efficient standardization and the development of minimal viable products.

## 1. Introduction

Holographic communication has opened up a new frontier for communication engineers and researchers. This new method for remote correspondence promises to fulfill the need for realistic and immersive human-to-human interaction with the help of virtual reality (VR), augmented reality (AR), 6G, artificial intelligence (AI), and other disruptive technologies. The field of holographic technology has seen extensive research and development, particularly focusing on integral areas such as data acquisition, high-bandwidth/low-latency transmission, and real-time processing and rendering of holographic data. Despite these advances, HTC remains in its infancy, and the market still lacks commercially available products that have achieved widespread consumer adoption. This situation necessitates continued research and development to facilitate the creation of more minimal viable products (MVPs). Ultimately, we aim to streamline this process, making it easier and faster to engineer a product that can successfully break into the market.

Significant advancements in HTC are evident in the wide array of sophisticated techniques now available. From three-dimensional data acquisition techniques employing RGB and RGB-D sensors [1,2,3] to intricate processing procedures for holographic data, including three-dimensional (3D) object reconstruction [1,4,5], pose estimation [6,7], and activity prediction [8,9], the field is progressively evolving. In addition, the introduction of 5G [10,11,12,13], the anticipated emergence of 6G [14,15], and the application of AI technologies [16,17] will together enable the efficient transmission of voluminous data with minimal latency, potentially optimizing network performance and enhancing overall transmission efficacy. Lastly, rendering and AR/VR visualization have seen considerable advancements, with state-of-the-art methods like ray tracing [18,19] and neural radiance fields (NeRF) [20,21] offering increasingly realistic and immersive experiences. Despite all this work, however, little effort has been made to better the interoperability and integration of these approaches within the boundaries of an HTPS. Moreover, there is a pressing need to design a more effective environment that encourages the development and evaluation of new methods, designed with integration into a unified HTPS in mind from the outset.

In this paper, we analyze key HTPS developments, focusing on their technical successes and limitations. ’Holoportation’ [3] and the platform by Tonchev et al. [22] are crucial milestones but face challenges related to high-end hardware dependencies and inflexible physical-unit-based architectures. These technological and structural issues shape our proposal, guiding us to enhance their benefits while avoiding known pitfalls. These works have notably pushed HTC towards wider consumer adoption. For instance, ’Holoportation’ has spurred the creation of many open-source libraries, supporting the widespread use of the Hololens headset. Similarly, the LiveScan3D system [23] open-sourced its data acquisition system code, facilitating the Kinect V2 sensor’s incorporation into HTPS development. These examples underline a trajectory toward democratizing HTC technology, a path we aspire to further carve with our proposal.

In response to these identified challenges and gaps in the field of HTC, a novel system architecture is proposed, one that consolidates research, development, evaluation, and integration processes for HTPSs, enabling a more focused and independent approach to module development. This proposed architecture lays the foundation and outlines the essential architecture blocks (ABs) required to establish a structured, efficient, and interoperable platform for the rapid development of cutting-edge solutions. At its core, the system we propose constitutes a modular, layered platform that realizes the previously mentioned architecture. This platform provides a research-friendly environment for the seamless development, integration, and evaluation of state-of-the-art methods across the different layers of an HTPS from data acquisition to rendering. Here, the term ’module’ refers to the practical implementations of the ABs within the platform. A distinctive feature of our platform is its emphasis on flexibility, where modules within each layer can be interchanged with ease, embodying the adaptability designed into the architecture.

As a demonstration of this architecture’s capabilities, we have successfully implemented several modules, including those associated with data acquisition, processing, immersion, and management layers, all within our platform. A comprehensive outline of these implementations, as well as proposals for future exploration of unimplemented modules, will be detailed in the following sections. Our key contributions include the definition of a versatile architecture for HTPS and the design of a robust platform based on said architecture, which actively encourages research and development in HTC. This structured yet flexible system bridges various research and development efforts, serving as a sandbox for testing new and emerging technologies. A significant aspect of our contribution also lies in the development and integration of several modules within the system. Our initial results show that these modules function cohesively, potentially speeding up the advancement and wider application of HTC technology. These findings are further explored in subsequent sections of this paper, along with the concrete implementation of the different components of the platform.

This work is a significant component of the HOLOTWIN Project, an initiative focused on enhancing the research and innovation capacity of the Technical University of Sofia in HTPS. The project facilitates a comprehensive knowledge transfer process from distinguished institutions: Aarhus University (AU), the University of Surrey (UNiS), and Hellenic American University. The system we have designed reflects HOLOTWIN’s goals, and we believe it is a great example of what we can achieve together. As a versatile and interoperable platform, it furthers the project’s objectives by encouraging research and innovation in HTC, leading the way towards the accelerated development and wider adoption of this technology.

## 2. Background

The field of HTC has experienced remarkable growth in recent years, with potential applications in fields like education, healthcare, and entertainment, among others. In this section, some of the notable HTPS developments that have emerged over the years will be explored, grounding our discussion in the historical context of this rapidly evolving field. Additionally, we will reflect on our previous work, capturing lessons learned and innovations that have informed our current endeavors. To fully appreciate the scope of our proposed HTPS architecture, it is crucial to understand the basic concepts, historical progress, and current advancements in this field. This background information will provide a broad perspective, setting the stage for a deeper dive into the specifics of the HTPS.

### 2.1. Foundations of HTC: Theoretical, Architectural, and Logical Perspectives

#### 2.1.1. Deployment Scenarios

In this section, we aim to provide an idea of the scope and adaptability of HTC. Akyildiz and Guo [24] outline three deployment scenarios that demonstrate the potential applicability and versatility of HTC. These scenarios and their concrete applications are exhibited in Table 1 alongside their specific requirements.

#### 2.1.2. AI, Integration, and Future Prospects

In Ref. [14], Strinati et al. lay out an ambitious roadmap for the evolution of internet communication architecture with a specific focus on holographic communication. They highlight the need to develop an internet architecture that coalesces various resources such as communication and computation within a single framework to support deterministic services. The paper details how a new data plane would be required to dynamically adapt to different operating modes, paving the way for effective HTC. Both Refs. [14,25] emphasize the significance of adopting a holistic management strategy for the C4 resources (communication, computation, caching, and control). The efficient orchestration of these resources is essential for the successful implementation of a cohesive HTPS. In such systems, computational resources play a critical role that is at least equal to, if not more than, that of communication resources. This is due to the extensive processing needed to generate, encode, and decode the holographic data. Therefore, the joint optimization of these resources is deemed critical to achieving low-latency, high-efficiency HTC. Strinati et al. pinpoint end-to-end (E2E) delay as a performance indicator in HTPSs due to it encompassing both communication and computation time.

Petkova et al. [25] identified a gap in current HTPSs, emphasizing that the present technology may not sufficiently meet rising demands. Rather than solely advancing technology, they propose the integration of intelligent methods into HTC systems, leveraging computer vision (CV) and AI to address these challenges. Central to their vision is the development of an HTPS that combines technological advancements with strategic AI and CV processing, aiming for efficiency, timeliness, and cost-effectiveness. Their guiding principles are lightweight processing, ultra-low-latency transmission, scalability, and high realism in avatars.

While the work in [25] focuses more on the data-processing potential of AI, Strinati et al. [14] also place AI at the heart of the transformation towards a holistic internet infrastructure. They envision distributed AI algorithms playing a key role in multiple aspects of this new network, such as the self-optimization of network resource allocation and the development of context-aware virtual intelligent assistants. These AI-driven tools are seen as instrumental in making HTC more personalized and efficient, particularly in learning from and predicting user behavior.

The potential utilization of AI and edge intelligence in HTC has been studied in [24]. As stated by the authors, AI can foster dynamic network adaptability, refining network topology in line with user service demand, scheduling maintenance, and facilitating hardware and software upgrades. Moreover, AI can play a significant role in real-time network control, encompassing aspects like network monitoring, adaptive routing, and network slicing. On the other hand, edge intelligence is positioned to have a significant influence on HTC networks, according to [24]. The conventional practice of relying on cloud-based solutions for aggregating sensor data and executing point cloud compression can lead to substantial latency due to the geographical separation between user and cloud. To optimize this, computation resources need to be brought as close as possible to the end user, a strategy promoted by the deployment of mobile edge computing (MEC). MEC is already an important component of 5G, but its role in managing and integrating communication and computation resources is expected to be even more crucial in a 6G environment.

Diving deeper into innovative strategies powered by AI, Ref. [14] mentions the introduction of semantic inference algorithms and semantic communication strategies into HTC [26]. These strategies are designed to embed knowledge representation into communication frameworks, thus optimizing holographic data transmission. Strinati et al. suggest that even though semantic aspects were deemed irrelevant in Shannon’s classical information theory (CIT), the basis of modern communications, the next step in the evolution of communications would involve integrating these semantic aspects. By doing so, we could push beyond the limitations of CIT, particularly with regard to holographic communication, a concept also outlined in [10]. Incorporating AI into data compression effectively shifts the burden from communication resources to computation. Such a shift optimally calls for computation resources to be positioned as close to the end user as possible. This philosophy is endorsed by the deployment of MEC.

#### 2.1.3. Architectural Foundations

In this section, we turn our attention to the logical structure that is necessary for a functional HTPS across the deployment scenarios detailed in Table 1. Delving from an overarching view to a more detailed perspective, Akyildiz and Guo [24] provide a generic HTPS architecture that consists of three components: source, network, and destination. Petkova et al. [25] reinforce this structural perspective by stating that, at its core, any HTPS requires multi-sensory data capture and reproduction, data processing, and data transmission. Notably, these steps also align closely with the four distinct layers of the HTPS architecture proposed in the next section.

- **Source:** The source is equipped with a diverse range of sensors capable of capturing an array of sensory data, spanning from visual and auditory to haptic, olfactory, and potentially gustatory signals. Following data capture, the source undertakes intricate processing tasks to transform the raw sensory data into a more structured and meaningful format. This includes generating point clouds from multidirectional images, synchronizing disparate multi-sensory data, and compressing the holographic data to mitigate network load. Finally, the source employs specialized HTC networking protocols to transmit the processed data packets.
- **Network:** The network, whether wireless or wired, serves as the robust backbone of the HTC system, shouldering the responsibility of transmitting voluminous source data with assured and bounded end-to-end latency. This involves harnessing a myriad of cutting-edge technologies, such as software-defined networking (SDN), automatic network slicing, and content caching. Emerging technologies like semantic communications, deterministic networking, novel transport layer solutions, and latency control algorithms are also deployed to fortify the network’s robustness.
- **Destination:** The destination component of the HTC architecture is designed to receive and reproduce data. It incorporates various actuators (e.g., olfaction and gustatory) to faithfully recreate the original environment captured at the source. Synchronization between the holographic display, actuators, and speakers is also ensured. Beyond this, the destination component proactively rectifies errors and augments the quality of holograms and multi-sensory media using super-resolution technologies. It also employs motion sensing to adaptively request services, further enhancing the quality of the output.

The challenges outlined in [25] aid in identifying distinct logical entities within the structure, each tasked with addressing a particular challenge. One of the primary challenges in an HTPS is spatially aware data acquisition. Petkova et al. delve into the complexity of capturing data across an array of senses: visual, auditory, tactile, olfactory, and gustatory. This necessitates the deployment of sensors that are not just spatially distributed across a region but also tailored to capture the diverse sensory experiences humans undergo. In cases involving multiple visual data sources, synchronization becomes imperative. Petkova et al. underscore the importance of this preliminary synchronization, pointing to the need for a dedicated entity focusing on aligning data acquisition equipment. Once the multi-sensory data are acquired, they enter the processing pipeline. This pipeline is multi-faceted, tackling tasks like 3D visual data reconstruction, multi-source stream synchronization, and data compression. Subsequent to processing, the heterogeneity of the data demands their transmission over an efficient network channel, which Petkova et al. identify as a crucial node in the HTPS architecture. Finally, the data are prepared for user consumption, with the visual segments rendered via a 3D data rendering entity, while other multi-sensory information is rendered through a specialized multi-sensory data reproduction entity.

There exist multiple strategies to meet the demands of an HTPS. For instance, Aslam et al. [27] propose a layered architecture for the metaverse, providing a unique perspective on how to address the multifaceted requirements for this type of virtual environment. Their architecture consists of the following layers:

1. **Physical Space:** Central to the metaverse, this encompasses devices like VR headsets and servers, bridging the human and digital realms through a robust communication infrastructure.
2. **Social Space:** The metaverse enriches traditional social networks, transforming them into immersive digital ecosystems powered by VR, from gaming to virtual commerce.
3. **Virtual Space:** Acting as an interface between the physical and the digital, it hosts virtual arenas for education, commerce, and individual digital avatars, all fueled by cutting-edge rendering technology.
4. **Metaverse Engine:** This core segment processes data from both real and virtual domains, orchestrating interactions among digital entities and enabling computations for 3D models.

In observing this layered architecture, one can discern a user-centric viewpoint. This approach positions the end-user at the core, emphasizing their experience and interactivity within the metaverse. However, when considering the intricacies of modeling an HTPS, a data-centric approach may be more appropriate, since a user-centric model can sometimes overlook certain functionalities that are crucial in addressing specific challenges inherent to HTC.

Petkova et al. propose the RADI platform in [25], focusing on realistic avatar reconstruction, adaptability for multiple users, and high-fidelity dynamics and integration. The architecture is split into two: the server part, centralizing control and data, and the user part, representing participants. The user process involves capturing via sensors, body extraction, and AI-assisted shape optimization. The server manages communication and data fusion in both the virtual meeting environment (VME) and the physical meeting environment (PME). The proposed platform offers a clear direction, suggesting that for an effective HTPS to leverage AI’s benefits, it should be inherently designed to seamlessly integrate such advanced technologies.

Tonchev et al. have proposed a mixed-reality telepresence system (MRTS) architecture in [22], that is physical and hardware-centric in context. They have systematically identified and detailed the core blocks (as shown in Table 2) of the architecture, describing their specific responsibilities. This is all done to ensure the MRTS meets its required operational criteria. The framework they provide emphasizes the importance of each component in achieving a cohesive and effective telepresence system. Additionally, Tonchev et al. discuss the potential role of cloud computing, envisioning it as a powerhouse that could potentially centralize many of the system’s computational functions.

While the aforementioned architectures each present their unique vantage points in understanding and implementing HTPSs, they also demonstrate distinct limitations. The architecture proposed in [27], for instance, while innovative in its layered approach and user-centric viewpoint, remains somewhat tangential to the nuanced and intricate subject of HTC. The focus on a user-centric model, although essential for user experience, can sometimes obscure or marginalize the underlying technological challenges that are paramount to HTC’s efficacy. The RADI platform emphasizes the distribution of functionalities between server and user sides and distinguishes real-time requirements. While it provides an overall structure for HTPSs, it does not explicitly define certain key blocks responsible for specific tasks, potentially leading to operational gaps. Meanwhile, the architecture delineated by Tonchev et al. leans heavily towards a hardware-centric model. While this approach offers clarity in understanding each component’s role and operational mechanics, it also introduces rigidity, potentially stifling versatility and adaptability—critical elements for a system striving to stay abreast of rapid technological advancements. Nevertheless, both Refs. [22,25] define core requirements for the HTPS architecture upon which future innovations will be anchored and adapted.

### 2.2. Case Studies and Implementations of HTPS

In this section, we transition from the theoretical and architectural foundations of HTC to an exploration of its practical realizations. When examining the implementation of HTPSs, two distinct perspectives emerge that offer valuable insights into the complex dynamics of these systems. The first approach involves a layered analysis, wherein the implementation is dissected into distinct layers, each with its specific responsibilities and functionalities. This stratified perspective allows for a detailed examination of each layer’s role and how it contributes to the overall system. The second approach, on the other hand, advocates for a holistic examination of the HTPS implementation. Instead of isolating the layers, this perspective emphasizes the interconnectedness of different functions, which may span multiple layers. It provides a broader view, focusing on how these functions interplay and cooperate to enable seamless holographic communication. Throughout this section, we will delve into both of these approaches, highlighting the unique insights they offer into the practical implementation of HTPSs.

#### 2.2.1. Data Acquisition

Hooft et al. [28] address the source side of their proposed architecture with a comprehensive look into the methods of content capture and representation as well as content encoding. Three-dimensional objects are captured through an array of camera setups or LiDAR based cameras, enabling the use of image-based, volumetric media-based, or mesh-based solutions for representation. Notably, the choice of representation directly influences the perceived quality and memory requirements.

The ’Holoportation’ system, proposed in [3], utillizes eight camera pods around a room to capture the full physical environment. Each pod has two near infra-red (NIR) cameras, a color camera, a diffractive optical element, and a laser for projecting a pseudo-random pattern. The camera pods generate a color-aligned RGB and depth stream using stereo matching. The generated depth streams are essential for building 3D models of the scene. Several techniques were considered for this, including structured light, time-of-flight, and depth from stereo, but each presented issues. The final solution was to use active stereo for depth estimation, which helped estimate depth even for textureless surfaces.

Both Refs. [22,23] utilize commercial data acquisition platforms in their implementations. In Ref. [23], Kowalski et al. presented ’LiveScan3D’, a data collection system using multiple Kinect v2 sensors. Each Kinect v2 is connected to its own computer, which is linked to a central server. Given the constraints of the Kinect v2 Software Development Kit (SDK) and the limitations of PCI-E 3.0 motherboards, this client-server structure ensures synchronized frame capture and minimal network traffic during recording. Data are initially stored on the client computers and, post-recording, transmitted to the server for merging. Both systems adopt a similar strategy for the data acquisition infrastructure, where sensors are connected to a distributed (relative to the local hub) computing unit. This computing unit is responsible for initial data pre-processing before the aggregated data are further processed in a local hub. This design inherently concentrates computational resources, as illustrated in Table 2, where the local controller and mixed-reality server are allocated to a unit with substantially higher computational capacity.

#### 2.2.2. Processing

In Ref. [3], data acquisition results in large amounts of voxels, which are later transformed into a mesh using the marching cubes algorithm. While the mesh representation lowers the data volume, the meshes are further optimized by reducing the position data size through quantization. Additionally, the mesh indices portion is compressed using LZ4 to efficiently decrease the overall data size. Color imagery, on the other hand, only requires transmission from regions of interest; thus, non-foreground regions are set to a constant background color value prior to transmission, effectively reducing data size. All those processing steps were accelerated by utilizing graphical processing unit (GPU) computing, as it enables parallel processing of image data.

Hooft et al. delve into the intricate balance required in HTC systems between compression needs and real-time application constraints. They accentuate the necessity for efficient compression techniques to handle the significant storage and transmission demands of volumetric media. They detail an encoding technique [29] that capitalizes on the correlation between successive point cloud frames, utilizing an octree structure for efficient spatial representation and compression, alongside the Moving Picture Experts Group (MPEG) video-based point cloud compression (V-PCC) [30], which offers notable lossy compression ratios between 100 and 500. Concurrently, they acknowledge the problems derived from the extensive encoding time these methods demand, which is particularly critical in real-time applications like HTC. To mitigate this, they propose the concept of culling, or the removal of redundant data, to expedite the processing time, a strategy that necessitates further exploration.

While Refs. [3,28] focus on processing with the goal of lessening the burden on communication resources and bandwidth, Ref. [23] highlights an alternative, yet equally vital, aspect of processing. A key technical challenge addressed was the unique coordinate systems generated by each Kinect v2. Calibration is utilized to anchor the sensors within a global coordinate framework. Concurrently, to reduce data noise, a filtering method was integrated. Data points are measured against their ’n’ closest neighbors, and those exceeding a threshold distance ’t’ from their furthest neighbor are classified as noise. This frame transformation and filtering are conducted on the individual client machines, streamlining the system and facilitating real-time filtering. It is important to acknowledge that, while data compression serves as a means of offloading the big-data burden from communication resources onto computational resources, there are indispensable processing steps—such as calibration, noise filtering, texturing, pose estimation, and reconstruction—whose primary objective is to enhance holographic data quality and, consequently, improve the quality of experience (QoE). These processing steps, essential for achieving high fidelity in holograms, demand computational resources that are at least commensurate with those required for data compression.

#### 2.2.3. Transmission

The system proposed by Escolano et al. [3] illustrates a more focused and simpler application of holographic data transmission techniques. Their system employs a direct Transmission Control Protocol (TCP) wire format for data transmission, accommodating 5–6 viewing clients between capture locations via a single 10 Gbps link. The system’s average per-frame transmission size is reduced to 1–2 Gbps for a 30 frames per second (FPS) stream. Compressed frames are sent between capture sites and each active rendering client. For larger broadcast scenarios, such as music or sports performances, additional processing might be needed.

Building upon this straightforward example, the discussion now transitions to more complex and versatile approaches to data packaging and delivery, as outlined in [28]. Their work illustrates the widespread use of HTTP adaptive streaming (HAS), which enables a variety of quality representations to be accessed by the client based on factors like bandwidth, buffer status, and user preferences. The paper highlights the role of application and transport layer optimizations in streaming, such as UDP-based protocols like WebRTC and the emerging HTTP/3 (based on QUIC) that bypass TCP’s in-order delivery requirement. They also underscore the careful management of these protocols for high-bandwidth 6DoF content, citing the joint optimization of application and transport layers in Palmer et al.’s approach [31]. This includes the use of an adapted QUIC protocol that ensures the reliable delivery of key frames and fewer rebuffering events, a strategy they suggest can be applied to volumetric media. The discussion further considers the potential of intelligent network components like multi-access edge computing for strategic content caching, swift decision-making, and the utilization of strategies like network coding, network slicing, and extensions of dynamic adaptive streaming over HTTP (DASH) like server and network-assisted DASH (SAND) for improved client decisions on rate adaptation and content sourcing.

In exploring the application of WebRTC and QUIC protocols for HTC, a thorough analysis of their functional capacities is essential. WebRTC [32] enables straightforward, plugin-independent, real-time multimedia exchanges, navigating through historical challenges such as the need for specialized networks and software. WebRTC utilizes a signaling channel to establish a peer-to-peer data path between applications. Nevertheless, while it offers communication across diverse platforms via its interfaces, it wrestles with issues like the undetermined future of codec integration. The absence of integrated codecs suitable for managing holographic content in WebRTC, coupled with the uncertain future application of codecs, presents a significant obstacle to its utilization in a consumer-oriented HTPS. This impediment constrains its deployment in the development of MVPs, thereby limiting the exploration and realization of its potential in consumer-facing HTC solutions.

QUIC [33] collaboratively functions with HTTP/2’s multiplexed connections, facilitating multiple data streams to independently traverse to all endpoints, unhindered by packet losses in disparate streams. This stands in contrast to the potential head-of-line-blocking encountered by HTTP/2 on TCP during packet delays or losses. Furthermore, QUIC aspires to minimize connection and transport latency while also accurately estimating bandwidth in each direction to avert congestion. By relocating congestion control algorithms to user space on both endpoints, QUIC strategically places itself to rapidly evolve these algorithms. Importantly, QUIC and WebRTC are not mutually exclusive; QUIC can be utilized as a transport layer protocol for WebRTC’s data path, allowing the strengths of QUIC to be leveraged in WebRTC applications where pertinent.

In the practical application of WebRTC for HTC, optimal-use cases likely align with one-to-one scenarios, particularly given the substantial computational demands inherent to scene fusion in many-to-many communications, where each client must independently process and aggregate multiple VMEs into a coherent holographic representation. Thus, for many-to-many scenarios, a more centralized approach would be more beneficial. Meanwhile, the study explored in [34], where DASH is utilized atop the QUIC protocol, reveals a pathway toward efficaciously meeting HTC’s high-interactive requirements. To overcome these challenges, Yen et al. [34] propose a method that leverages the QUIC protocol, optimizing 360° video DASH streaming with its intrinsic secure communications, multiplexed streams with prioritized schedulers, and reduced latency. Thus, it enables the rapid transmission of urgent content at high priority when viewers adjust their orientation, thereby maintaining an immersive and uninterrupted user experience while preserving the simplicity of DASH streaming.

#### 2.2.4. Immersion

On the client side, Hooft et al. discuss the key roles of rate adaptation and viewport prediction. In volumetric media scenes, spatial segmentation becomes critical, and the client is tasked with deciding the quality of each object in a scene based on the available bandwidth, video bitrate, buffer status, bitrates and locations of objects within the scene, user focus and position, and spatial quality differentiation. Next, they explain that viewport prediction, which accurately predicts the user’s position and focus, is crucial in allocating bandwidth to the most significant regions within the scene and compensating for future user movement. They also consider the impact of content decoding, noting that while the V-PCC encoder offers superior visual quality, it cannot be used in real-time on regular hardware. Finally, the paper addresses content rendering, primarily performed through Unity and Unreal Engine, as is the case in [22]. They highlight the computational intensity of rendering, pointing out that current hardware requires significant time to render complex objects at high frame rates. Therefore, a proposal for using MEC resources is made of offloading rendering tasks to the network, although this approach necessitates real-time communication with the client.

In a practical application of these principles, Escolano et al. [3] design their system to address these very challenges. Recognizing the high computational cost of rendering detailed, high-quality 3D models on untethered VR or AR head-mounted displays (HMDs), their system offloads this rendering task to a dedicated desktop PC. This remote rendering approach allows for a better frame rate, less perceived latency, and the conservation of battery life on the device. The rendering PC is connected to the HMD via Wi-Fi and constantly receives the user’s 6DoF pose, predicts a headset pose at render time, performs scene rendering, and transmits the stream to the HMD for display. Any misprediction in the user pose is compensated for by rendering into a larger field of view, allowing for small rotational errors.

#### 2.2.5. Holistic Perspectives

The approach in [28] presents an architecture that is arguably more generalized than the ones proposed by Escolano et al. in [3] and Tonchev et al. in [22]. This architecture is centered around QoE, aiming to facilitate a use case where multiple people can generate a virtual scene to be experienced by users through an HMD. Its primary components include source capture, network delivery, client-side user movement tracking and focus, and service quality evaluation. This architecture underscores user experience assessment and scalability in delivery through a content delivery network, marking its intent for widespread user engagement.

In contrast, the ’Holoportation’ system by Escolano et al. and the platform proposed by Tonchev et al., while representing significant achievements in their own rights, exhibit limitations rooted in their design philosophies. Notably, the Holoportation system is heavily reliant on high-end computational equipment, which poses challenges for widespread adoption due to the high costs and limited accessibility of such equipment. In terms of architecture, both systems are designed with structural blocks (e.g., sensor controllers) that are implemented as physical units, not merely as logical entities. This implementation choice rigidifies the architecture and makes the incorporation of new technologies or the adaptation of system capabilities a significant challenge. For example, migrating some processing tasks to the cloud—a change that could be necessitated by the implementation of next-generation processing techniques for holographic data—would require a substantial overhaul of these systems due to their fixed, physically defined structure.

## 3. Proposed Architecture Overview

In this section, we will delve into the details of the proposed architecture, focusing on its layers and their structure. Our discussion will cover the practical aspects of this architectural design, demonstrating its versatility and intrinsic modularity. The key idea behind our design approach has been to encourage flexibility and ease of integration, ensuring that different elements can work harmoniously together while allowing modules within a layer to be easily swapped or replaced, further enhancing the platform’s adaptability. As we move forward, our focus will shift toward the specifics of implementing various methods within the platform, aiming to offer practical insight into their applications. We believe this approach will contribute to a more adaptive and versatile holographic communication platform, one that is ready to meet the demands of an ever-evolving technological landscape.

**Figure 1:**
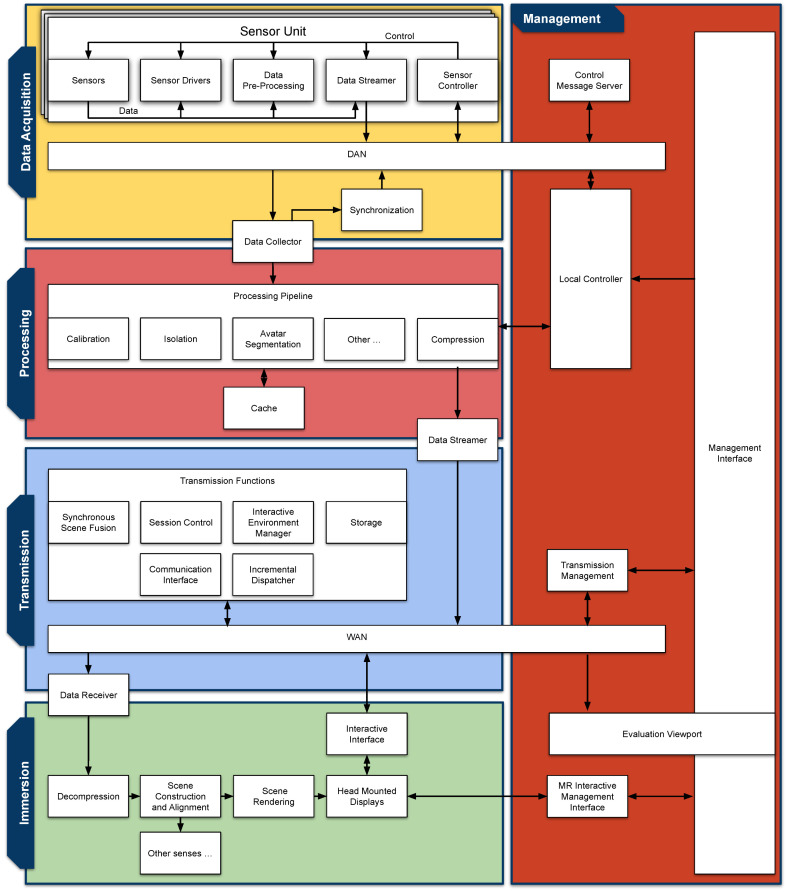
A layered model of the proposed architecture.

To provide a clear overview of our proposed HTPS architecture, we have conceptualized a layer diagram (Figure 1) that encapsulates its various components. The diagram outlines four core layers: Data Acquisition, Processing, Transmission, and Immersion, each dedicated to a specific task in HTC.

1. The Data Acquisition layer is responsible for the initial collection of raw sensory data, acting as the entry point into the platform.
2. The Processing layer receives these data and applies various algorithms and techniques to manipulate and refine them. Its role is to transform the raw sensory input into a virtual scene: a format of meaningful and usable information.
3. The Transmission layer manages the secure and efficient delivery of the processed data to the all nodes in the session.
4. The Immersion layer is where the transmitted data are transformed into an immersive, user-centric experience. While visual rendering is the primary function, this layer also has potential to engage other senses, crafting the final holographic output. It is here where the scene is rendered and user interaction is considered.

Beyond the four primary layers, we have also introduced a Management plane. This component is not a separate layer but rather intersects with each primary layer. The Management plane is the arena for system-wide coordination and control, providing the capacity for hands-on experimentation with different module configurations. By enabling human-led management and evaluation, we aim to streamline research efforts and accelerate development.

As illustrated in Figure 1, each layer contains multiple ABs, which represent the essential elements required for a functional HTPS. It is worth noting that some of these ABs may be optional, depending on specific use cases. However, the value of the proposed architecture lies in the ability to implement each AB with a variety of methods; we refer to these specific implementations as ’modules’. Each AB can be implemented through different modules, allowing for a high degree of flexibility and adaptability. For instance, the ’Data Streamer’ AB within the Transmission layer could be implemented using different streaming protocols, such as WebRTC or QUIC. These options are not mutually exclusive but can be selected and interchanged based on the requirements of a particular scenario or application.

### 3.1. Data Acquisition Layer

The Data Acquisition layer is the entry point of our architecture, where the initial sensory data are collected. Data acquisition happens through multiple sensor units dispersed in the environment, each potentially equipped with various types of sensors. These sensor units are interconnected via a data acquisition network (DAN), providing an infrastructure for the smooth flow of data from the sensors to a central point, the data collector. The data collector serves as an aggregator of the sensor data, preparing it for further processing in the subsequent layers.

1. **Sensors:** The actual hardware that captures data, such as radiometric sensors, depth sensors, microphones, etc.
2. **Sensor Drivers:** Software components that control and manage the sensor hardware. They facilitate communication between the system and the sensors, enabling the retrieval of the data from the sensors.
3. **Data Pre-processing:** Processing of the raw data captured by the sensors. It might include compression to minimize the data size, transformation to a more convenient format, filtering of noise, or other operations that prepare the data for subsequent processing stages.
4. **Data Streamer:** This element ensures the data from the sensor units are transferred successfully to the Data Collector via the DAN.
5. **Sensor Controller:** Provides the control functionality for the sensor unit. It coordinates the operations of multiple sensors within a unit, interpreting and enforcing commands from the Management plane.
6. **Data Collector:** The interface between the Data Acquisition and Processing layers. It gathers all sensor data in a centralized location, enabling further processing.
7. **Synchronization:** Ensures that the data streams from all sensors are properly aligned in time. Such synchronization is key to preserving the coherence of the multi-sensor data.

### 3.2. Processing Layer

The Processing layer is responsible for the transformation of raw sensor data into meaningful information in the form of a virtual scene. The layer features a Processing Pipeline, a Cache, and a Data Streamer.

1. **Processing Pipeline:** The main venue for algorithmic manipulation of the sensor data. It consists of several processing steps, such as calibration, isolation, segmentation, etc. The pipeline can be partly distributed; that is, some processing steps could be situated on-site, close to the data acquisition equipment, while others may be cloud-based. This allocation depends on factors like latency requirements, computational load, and resource availability.
2. **Cache:** Serves as temporary storage for frames, scenes, and other types of data, allowing for efficient access during the processing phase. This is particularly useful for processing algorithms, where the output depends on previous outputs or inputs, effectively supporting operations that involve temporal dependencies or require a historical context.
3. **Data Streamer:** The gateway between the Processing and Transmission layers. After the scene is fully constructed within the Processing Pipeline, the Data Streamer is tasked with streaming it into the WAN in order for it to be distributed to the other session participants. The Data Streamer plays a pivotal role in enforcing the communication protocol and managing the transit of data across the WAN, ensuring that the virtual scene is securely and efficiently transported to the Transmission layer. Depending on the transmission topology—centralized or decentralized—the Data Streamer utilizes the Communication Interface in the Transmission layer differently. In a centralized setup, it manages the upload of data to a centralized location, such as a cloud server, where all virtual scenes are aggregated before distribution to session participants. Conversely, in a decentralized model, the Data Streamer establishes peer-to-peer connections, managing direct data streaming amongst participants.

It is important to note that the processing pipeline is largely serial; that is, the output from one process is typically used as input for the next, forming a sequential data flow through the pipeline. This is not to say that parallelism is absent; individual processes can potentially execute parallel computations within their own implementation. However, the nature of the pipeline is such that executing two completely different processes concurrently often proves impractical due to the inherent data dependencies between stages.

### 3.3. Transmission Layer

The Transmission layer is where processed data are securely and efficiently distributed to all HTC session participants. This layer contains two ABs:

1. **Transmission Functions:** This AB serves as an aggregation of multiple vital functions, each specialized for the secure and efficient distribution of holographic content to all participants. It can operate in diverse environments, including cloud-based systems, and is designed for executing network-related tasks. The Transmission Functions AB consists of several sub-components:
  - **Communication Interface:** This serves as the entry point into the Transmission Functions framework, acting as the interface through which all participating sites connect to utilize the transmission functions.
  - **Session Control:** This component is responsible for the management of virtual sessions. Tasks include establishing and maintaining communication channels, confirming authorizations, and ensuring continuous connectivity among all session participants.
  - **Synchronous Scene Fusion:** This element aggregates the individual virtual scenes from each participating site. The result is a unified scene that combines the VME and participant avatars, which is then ready for streaming to offer an immersive experience to all participants.
  - **Incremental Dispatcher:** This AB scans the VME and avatars for significant changes and discards any redundant information. This ensures that only incremental updates are applied to the collective scene, rather than overhauling it entirely. This targeted approach significantly enhances the efficiency of data transfer and computation, leading to optimized resource utilization.
  - **Interactive Environment Manager:** This sub-component focuses on the interactive aspects of the VME. It aggregates the interactions of all participants to ensure a coherent interactive experience for all involved.
  - **Storage:** This AB operates as a data repository. It is optimized for high-speed data access and secure retention of various data types, including but not limited to holographic content, session metadata, and logs of user interactions.
2. **Data Receiver:** The interface between the Transmission layer and the Immersion layer. It accepts the prepared collective scene (VME and participant avatars) from the WAN, setting it up for rendering and interaction within the Immersion layer.

### 3.4. Immersion Layer

The Immersion layer acts as the endpoint where the user experiences the holographic communication. This layer contains the following sub-components:

1. **Decompression:** This AB handles the decompression of incoming data streams. Given the high data rates and the necessity of real-time performance in an immersive experience, data compression is a critical step in HTC. Consequently, decompressing these data accurately and swiftly is crucial in the immersion layer.
2. **Scene Construction and Alignment:** This component reinterprets the incoming data to prepare them for rendering or other sensory interfaces. The data representing different sensory experiences (such as sight, sound, etc.) are interleaved in the incoming data stream and need to be deinterleaved and reinterpreted before being forwarded to the corresponding sensory interfaces. Moreover, this block is also responsible for aligning the incoming VME and/or avatar data with the participant’s current PME.
3. **Scene Rendering:** Here, the geometric representation of the scene is converted into a visual representation that can be displayed on a device. This process requires high computational power as it needs to be performed in real-time for a smooth and immersive experience.
4. **Head-Mounted Displays:** This is the physical device worn by the participant, enabling mixed-reality (MR) immersion. It can either be an AR or VR device, depending on the specific use case.
5. **Interactive Interface:** This AB is responsible for managing the participants’ interactions within the VME. Serving as an interface, it collaborates with the Interactive Environment Manager in the Transmission layer. The module enables users to interact in an intuitive and seamless manner, enhancing the overall immersive experience. This may involve tracking user movements, interpreting voice commands, or other means of interaction, depending on the specific implementation of the system.

In essence, the Immersion layer is where the final transformation and delivery of the holographic data are carried out, enabling the user to be fully immersed in the interactive and high-fidelity MR experience.

### 3.5. Management Plane

The Management Plane is responsible for the overarching control and regulation of the entire HTPS. It contains several blocks distributed across the layers, serving specific functionalities.

1. **Control Message Server:** Existing within the Data Acquisition layer, this block is tasked with distributing control messages and commands to sensor units. It is linked to the DAN, where it receives these commands.
2. **Local Controller:** This AB, also within the Data Acquisition layer, keeps track of the states of each sensor unit, forwarding commands to them via the DAN. It can also extend control over the Processing layer, offering a broader range of command and control. It interfaces with the Management Interface, receiving commands that it translates into control messages for the corresponding sensor units.
3. **Transmission Management:** Residing within the Transmission layer, this block is dedicated to managing the Transmission Functions, offering remote control and configuration capabilities.
4. **MR Interactive Management:** Positioned within the Immersion layer, this management interface exists within the VME. It provides a way for a participant to manage the system, without breaking the immersion, thus allowing for interactive experimentation and evaluation of the system’s capabilities.
5. **Evaluation Viewport:** Also part of the Immersion layer, this AB enables direct system evaluation by the administrator. It provides a direct view of the virtual scene, allowing administrators to observe the effects of parameter changes, module swaps, and other alterations as they commence.
6. **Management Interface:** This AB, spanning all layers, serves as an interface for system administrators and researchers. It provides control over various system parameters, enabling the configuration of the HTPS according to specific requirements.

### 3.6. Summary

Table 3 provides a list of all the ABs along with a short and explicit summary of their responsibilities. The aim is to provide a non-ambiguous definition of every AB so that their position within the HTPS is clear.

## 4. Implementation

In this section, we focus on the practical application of our platform, with particular attention to the modules we have successfully implemented. However, it is essential to note that, while we have made significant progress, our platform is not entirely complete, and there are some gaps in the general implementation that we aim to address in future iterations.

We have established modules spanning the domains of Data Acquisition, Processing, Immersion and Management. The Data Acquisition modules include Sensor Drivers, Sensor Controller, and Data Streamer implementation. In terms of processing, our platform incorporates multi-view sensor calibration, scene extraction, and avatar segmentation. Immersion modules feature remote rendering for AR HMDs and interactive AR scene visualization. With regard to the Management plane, we have implemented a Control Message Server, Local Controller, Evaluation Viewport, MR Interactive Management Interface, and Management Interface. In the subsequent sub-sections, we will explore each of these components in greater detail, showcasing their functionality, interoperability, and potential areas for further development.

### 4.1. Physical Distribution of Architecture Blocks

**Figure 2:**
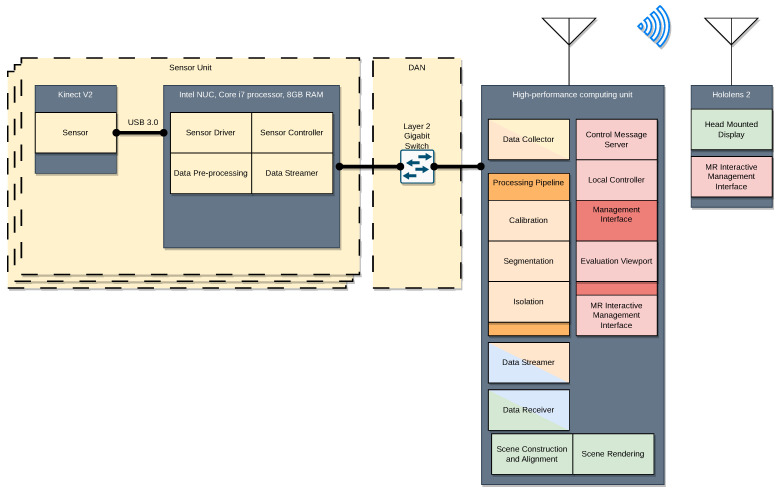
Physical layout of the platform implementation. The colors of the ABs correspond to the colors of the layers established in Figure 1.

The practical implementation of the proposed architecture involves optimally distributing the ABs across the units of some physical infrastructure. Figure 2 describes the physical layout of the platform implementation. The figure aims to provide clarity on the physical location of each AB within the hardware infrastructure of the HTPS. Our practical implementation utilizes the hardware infrastructure proposed by Tonchev et al. in [22], which is described in Table 2.

### 4.2. Data Acquisition

Our Data Acquisition infrastructure consists of three Sensor Units connected to a high-performance computing unit (HPCU) via a gigabit-switched local area network (LAN).

#### 4.2.1. Sensor Units

The proposed platform utilizes a combination of Kinect V2 sensors and Intel NUC5i7RYH mini personal computers (PC) to actualize the Sensor Units. The Sensor Driver, Sensor Controller, Data Pre-processing, and Data Streamer are all implemented on the NUC, as shown in Figure 2. The Kinect V2 sensors are connected to the NUCs via a USB 3.0 interface. We have implemented a Sensor Driver module, which utilizes the Kinect V2 SDK and the corresponding device drivers provided by Microsoft, to provide a transparent way of accessing the spatial capture capabilities of the sensor. This module provides an application programming interface (API) for point cloud acquisition as well as individual acquisition of the color and depth frames. The core functionality of this API is implemented in C++ and is later exposed in Python via a dynamic-link library (DLL). This approach is versatile, as it allows for both low-level and high-level programming (scripting). The Kinect V2 works at 30 FPS. These frames are stored internally until the Sensor Driver requests them. Consequently, the rate at which frames are perceived by the rest of the Sensor Unit is dictated by the rate at which they are requested by the Sensor Driver API (with an upper limit of 30 FPS). This allows a versatile frame rate that is adaptable to the service rate of the Data Pre-Processing and the rest of the system.

The Kinect V2 sensor generates color frames with a resolution of 1920×1080 pixels. The depth frames have a resolution of 512×424 pixels. Consequently, the captured point clouds consist of 512×424=217,088 points. Each point possesses positional coordinates, represented by 3 4-byte values, and a color, represented by 3 1-byte values. The Sensor Driver utilizes the Kinect SDK functionality to map the higher-resolution color images to the depth images. As a result, the lines between the Sensor Driver and Data Pre-Processing are blurred within our implementation. In fact, this mapping is the only pre-processing we perform.

The Sensor Controller module on our platform is implemented in Python. It provides an API that enables the following functionalities:

- Maintain a tree-like structure of the sensor devices available to the Sensor Unit.
- Register with the Local Controller.
- Provide a MQTT-based interface for the exchange of control messages.
- Execute query-based control commands.

Our implementation of the Sensor Controller module maintains a hierarchy of entities that can either be directories or Data Sources, where a directory is a collection of Data Sources without its own properties and functionalities. A Data Source consists of a Sensor Driver (with its corresponding physical Sensor), Data Pre-Processing, and a Data Streamer. It may optionally contain its own hierarchy of Data Sources. Each Data Source also contains a list of exposed properties that directly influence its behaviour. The aim of this hierarchical approach is to enable access to the properties of every Data Source, subordinate to the particular Sensor Controller, by defining the path to the source and the name of the property, similar to a file system. This construct allows for the independent, source-specific handling of data, where each Data Source pre-processes and streams its data independently. A positive by-product is the separation of data and control services, within the Data Acquisition layer.

The Sensor Controller module provides registration functionality, which enables each Sensor Unit to be registered with the Local Controller. This way, the Local Controller can keep track of all the Sensor Units in the DAN. The functioning of the Local Controller itself will be further examined in Section 4.6.2; however, a description of this registration process will be provided here. The Sensor Controller communicates with entities outside of the Sensor Unit via the MQTT protocol [35]. Our platform requires that all Sensor Units register with the Local Controller as a way of making the Local Controller aware of their existence. This registration process is carried out by publishing a message containing the Sensor Unit’s ID on a specific topic that the Local Controller subscribes to, followed by the Sensor Controller subscribing to a separate topic to receive future control messages. The MQTT communication requires a broker, which is implemented on the HPCU and is represented in Figure 2 as the Control Message Server.

The control messages are also executed by the Sensor Controller. Each message is in the form of a query with a specified path, property, and operation, e.g., ‘/path/to/datasource/property<–value’, where ‘–>’ and ‘<–’ correspond to the ‘Get’ and ‘Set’ operations. Regardless of the type of operation, the Sensor Controller returns a response. This response contains the value of the property after the execution of the control message. It is published as a common topic for all Sensor Units, to which the Local Controller is subscribed.

**Figure 3:**
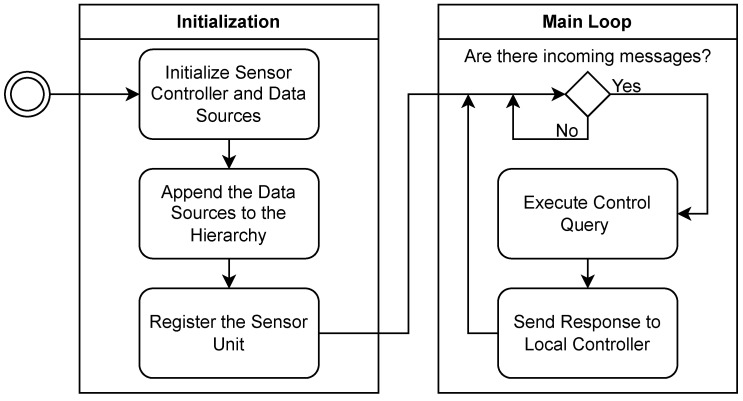
Sensor Unit program.

Keeping in mind the established processes within each Sensor Unit, we present a flowchart in Figure 3 to describe the program executed by every Sensor Unit.

#### 4.2.2. Data Acquisition Network

**Figure 4:**
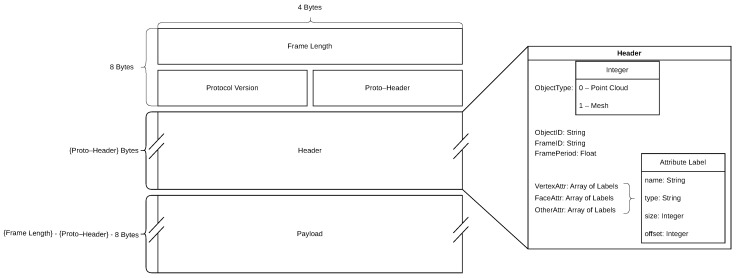
Frame format.

As mentioned in the previous section, each Data Source handles its data transfer independently. Data transfer is accomplished by a Geometry Streamer module on the Sensor Unit side. This Python-implemented module offers packetization functionalities for various types of geometric data, such as point clouds and meshes, among others. We will refer to the protocol data units as frames, since, in our case, each unit represents a frame captured by the Kinect V2. The structure of the assembled frames conforms to a custom application layer protocol, designed with the goal of supporting a variety of payloads related to HTC. The format of these frames is exhibited in Figure 4. Each frame can be divided into three parts: Meta-Header, Header, and Payload. The Meta-Header consists of the Frame Length, Protocol Version, and Proto-Header fields. Frame Length represents the complete length of the frame in bytes. Protocol Version indicates the version of the protocol. Finally, Proto-Header represents the length of the Header in bytes. The Header itself is a JavaScript Object Notation (JSON) object. This JSON object, exhibited in Figure 4, contains several fields generally describing the content of the payload as well as three arrays of attribute labels that give a detailed description of the individual attributes. More precisely, an attribute is a parameter that is used to describe the data points within the captured frame. Since our focus is on geometric data, we have categorized these parameters into three groups: vertex attributes, face attributes, and others. Vertex attributes describe the properties of vertices, and face attributes describe the properties of the faces of a mesh. The serialized attribute data are positioned within the payload, while the attribute labels (meta-data) are located in the Header. Each attribute label is itself a JSON object that specifies the name, type (integer, float, etc.), size, and offset of the attribute data.

In addition to its packetization functionalities, the Geometry Streamer module is further subdivided into sub-modules. Each of these sub-modules is tasked with handling the packetization processes at the underlying network layers. The sub-module operating at the Data Acquisition layer, for example, utilizes TCP sockets to facilitate data transfer to the Data Collector.

Each frame in the DAN is designated with the identification of the Data Source it originated from. More precisely, the frame carries this information in the Object ID field of the header. Our implementation of the Data Collector acts as a TCP listener, listening for connections from the Sensor Units. Upon arrival in the Data Collector, the frames are sorted into multiple queues corresponding to their Data Source. The frames are consumed by the processing pipeline once there is at least one frame in each queue.

### 4.3. Processing

This section emphasizes the algorithmic manipulation of the sensor data applied within the Processing pipeline, a crucial component of the Processing layer. These operations are executed on the HPCU, leveraging the capabilities of its GPU. This provides the capacity for parallel computation, thus expediting the data processing.

#### 4.3.1. Calibration

In the practical implementation of our platform, the PME is observed by the three Kinect V2 sensors, each capturing it from a unique perspective. To ensure a coherent environment reconstruction, we employ a calibration procedure, defined in a previous work [2]. Instead of employing a specialized calibration marker, the proposed method utilizes an object within the PME with semantic significance, specifically a table in our context. Numerous images of this object were previously gathered from diverse viewpoints and orientations, creating a dataset. The dataset was subsequently employed to train a Polygon-YOLOv5 [36] network for detecting the object’s feature points. These feature points exhibit a one-to-one correspondence across all viewpoints. They are further utilized to compute the rotation and translation parameters between the distinct perspectives.

**Figure 5:**
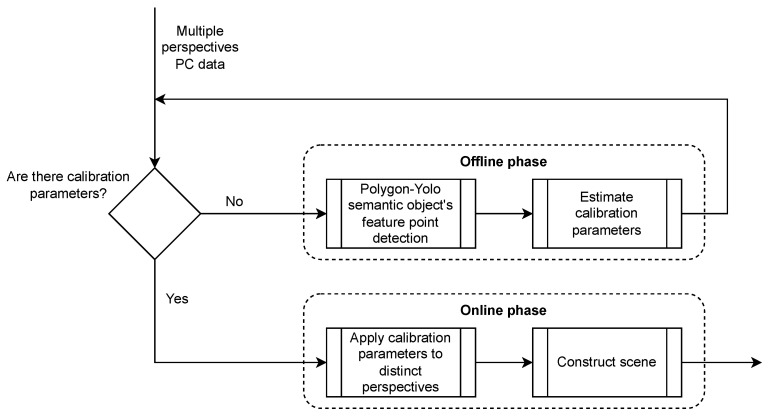
Calibration procedure.

The integration of this approach into our proposed platform uses Python and consists of two stages. First, during an offline phase, the 512×424 RGB images extracted from the point clouds captured by the three sensors are fed into the pre-trained Polygon-YOLOv5 network. It proceeds to identify the feature points of the semantically significant object, thereby enabling the calibration process. Second, during an online phase, the continuously incoming point clouds from the diverse viewpoints are aligned using the transformation parameters obtained in the offline phase. The calibration procedure is represented by the flowchart in Figure 5. To fulfill the real-time requirement of this step, the computational power of the GPU is utilized.

#### 4.3.2. Isolation

Within the Processing pipeline, we incorporate another processing step that focuses on extracting a scene from its ambient surroundings. This step exemplifies specific processing requirements that are essential in our particular case but may be dispensable in alternative scenarios necessitating different operations. In our specific scenario, we capture a controlled physical space defined by six walls forming a hexagonal cell called the ’Bee Cube’. However, it is important to note that extraneous data beyond the confines of the Bee Cube are also acquired. We employ a 3D scene-extraction algorithm previously introduced in a recent study [5] to extract only the Bee Cube from these data. In brief, the method seeks to identify objects with semantic relevance to the Bee Cube’s walls by employing a plane detection algorithm. An essential component of wall detection involves the computation of the wall surface normals and distances, which we call scene extraction parameters. Principal component analysis (PCA) is further employed to enhance the accuracy of these parameters.

**Figure 6:**
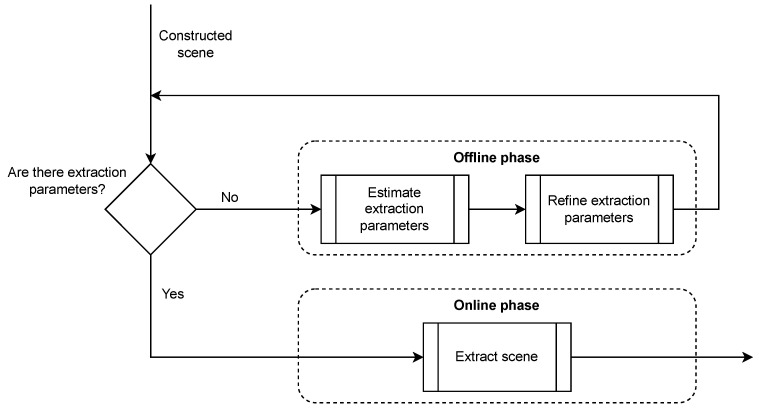
Scene isolation procedure.

The integration of this operation within the Processing pipeline of our HTPS platform is once again divided into two stages. This division is essential, as detecting the Bee Cube walls is not time-efficient and is not necessary to be performed in real time. In the offline phase, the wall surface normals and distances are calculated and refined in order to obtain the Bee Cube’s boundaries. In the online phase, they are applied to the aligned scene, thus isolating the Bee Cube from its surroundings. The flowchart in Figure 6 presents the scene isolation procedure.

This processing step is again performed in Python, harnessing the computing power of the GPU, particularly to optimize its real-time implementation.

#### 4.3.3. Segmentation

In the segmentation processing step, the system utilizes YOLOv8 [37], a state-of-the-art object detection algorithm, to segment out individuals from the frames received from various Sensor Units. Initially, the frames, containing both spatial and color information, are standardized for further processing. The YOLOv8 algorithm is then employed to analyze each frame, specifically searching for human figures based on a pre-defined confidence threshold.

During this analysis, if any segment passes the confidence score, a ’mask’ representing the person in the frame is generated. These masks essentially serve as high-confidence regions where a human is detected. For specified frames, these masks are resized to match the dimensions of the original frames. Using these resized masks, points from the spatial and color data are extracted to create a three-dimensional point cloud representation of each person within the PME. The system keeps track of performance metrics such as preprocessing, inference, and post-processing speeds for each frame, which can be utilized for performance evaluation.

### 4.4. Transmission

The Transmission layer of our architecture is streamlined to include only two main components: the Data Streamer and the Data Receiver. The former is implemented by the Geometry Streamer module, as detailed in Section 4.2.2. The latter is integrated within the Unity application, which will be elaborated upon in the following section. Both modules serve distinct but complementary roles: the Data Streamer handles the outbound geometry data, while the Data Receiver acts as the inbound endpoint and interprets incoming packets. It is worth noting that the Transmission layer’s communication occurs intra-system, as both the Data Streamer and Data Receiver are housed on the same physical unit.

Expanding on the Data Streamer, it employs the same TCP sub-module utilized for communication within the DAN. Additionally, the Data Streamer incorporates a specialized sub-module designed for streaming data to the Evaluation Viewport. Unlike the TCP sub-module, this additional component leverages the HTTP-based SocketIO protocol to provide a dedicated communication channel for real-time updates to the Evaluation Viewport.

### 4.5. Immersion

The functionalities within our platform’s Immersion layer are encapsulated in a Unity application, which is targeted for Hololens 2. This application performs remote rendering on the HPCU and wirelessly streams the rendered frames to the Hololens 2 via a Wi-Fi network. In return, the Hololens 2 transmits its 6DoF pose data back to the application. To achieve this, we heavily relied on the third version of the mixed-reality toolkit (MRTK) [38]. All Immersion layer blocks, except for the Decompression block, have been implemented; we found decompression unnecessary due to the intra-system nature of the transmission.

In terms of rendering, we employed Unity’s High Definition Render Pipeline (HDRP). This choice was driven by the HDRP’s superior quality output, especially since our rendering resources are virtually unlimited on the HPCU. Additionally, the HDRP facilitates efficient rendering of point clouds through its ComputeBuffer API, minimizing central processing unit (CPU) overhead. The data flow starts with frame interpretation in the Data Receiver, where attributes like point coordinates and color are extracted. These attributes are then used to reconstruct the point cloud within the Unity application, which is subsequently sent to the GPU for rendering.

For device-to-device communication between the HPCU and Hololens 2, we leverage Microsoft’s Remoting API. This API abstracts away the complexities of frame transmission, allowing us to focus on session establishment between the two devices. The receiving end-point on the Hololens 2 is facilitated by the Holographic Remoting Player, available on the Microsoft Store.

Lastly, we incorporated an interactive interface that allows users to manipulate the holographic scene by moving, scaling, and rotating it within their space. These operations are accomplished through hand-tracking and gesture recognition capabilities, again facilitated by MRTK 3.

### 4.6. Management

#### 4.6.1. Management Interface

The management interface for our HTPS is a web-based platform developed with a split architecture. The server-side logic, or backend, is implemented using FastAPI, while the user interface (UI) is built using React in conjunction with the Material UI module. SQLite serves as the database engine, storing both user settings and permissions.

The interface is designed for multi-user access, allowing for simultaneous connections and providing tiered user permissions. Each user can interact with a specific set of adjustable settings, represented by data fields that can contain different types of data, such as integers, strings, or booleans. The database stores these settings alongside user permissions, thereby ensuring that users can only manipulate settings they are authorized to access.

In addition to user management, the backend is capable of communicating with the Local Controller through the MQTT protocol. This feature enables real-time influence over the controller’s decision-making concerning command dispatch to the Sensor Units. The interface includes an operator dashboard, where the operator can initiate or terminate sessions and exert control over the sensor units’ behavior. This setup ensures a responsive and secure environment for both monitoring and management tasks, suitable for various users with different access levels.

#### 4.6.2. Managing Data Acquisition and Processing

The system relies on message-based communication, mainly using MQTT as a protocol for passing messages between the Management Interface, Local Controller, and individual Sensor Units. The program starts by setting up message queues for handling data and commands. It then subscribes to specific topics on the MQTT broker to listen for incoming messages from the web interface and Sensor Controllers. Each message is categorized by its origin and processed accordingly. When a Sensor Unit tries to register with the Local Controller, the controller will check if it is already aware of this unit. If not, it will create a new entry and manage its attributes like connection status, enabled state, and other operational parameters. The controller constantly checks for messages from the Sensor Units and updates their statuses based on these messages.

Additionally, the system has a state machine for its operational state, which can be ’Stopped’, ’Starting’, or ’Started’. The web interface can send commands to start or stop the system. When the ’Start’ command is received, the program checks the connectivity of all known sensors. If at least one sensor is connected, the system will attempt to enable all connected sensors and transition to the ’Starting’ state. The system will confirm that all sensors have been enabled before finally moving to the ’Started’ state. Similarly, when the ’Stop’ command is received, the system will attempt to disable all sensors and transition to a ’Stopped’ state only after confirming that all are disabled.

Lastly, the web interface can also request details about the connected sensors. In response, the local controller compiles this information and sends it back. The attributes of each sensor, like whether it is connected, whether it is an enabled state, and other operational modes, are sent in this information package.

#### 4.6.3. Evaluation Viewport

The Evaluation Viewport is a feature embedded within our web-based Management Interface, more specifically within the dashboard page, offering operators a unique vantage point to observe the VME. Instead of diving into the immersive environment, operators can evaluate its characteristics and behaviors from an external perspective, using user input to navigate through the 3D scene. This capability is particularly useful for assessing the effectiveness of system configurations, evaluating new processing algorithms, or simulating various scenarios to better understand their impact.

The viewport is seamlessly integrated into the operator’s dashboard and is engineered using the ThreeJS JavaScript library, a powerful tool for rendering 3D graphics directly in a web browser. This decision is driven by ThreeJS’s provision of a high-level abstraction of WebGL, facilitating effortless integration with React applications and ensuring efficient and high-quality visualization of the VME’s dynamic point cloud representation. Moreover, the decision is further substantiated by existing research on approaches for 3D visualization within web browsers [39,40]. Notably, ThreeJS also excels in rendering large quantities of points and managing complex scenes by optimally utilizing GPU capabilities, thereby significantly enhancing the evaluation process.

#### 4.6.4. MR Interactive Management Interface

Within our Unity application, we have developed a MR Interactive Management Interface module, leveraging the user interface features of MRTK 3 [38]. This specialized interface enables immersed users to initiate sessions and begin receiving holographic content. Additionally, it enables Hololens 2 wearers to establish a connection with the HPCU, facilitating the reception of rendered frames. This integration creates a cohesive and interactive user experience while maintaining high levels of computational efficiency and performance.

## 5. Results

In this section, we turn our attention to the evaluation of our implemented platform, delineating both quantitative and qualitative aspects of its performance and capabilities. It is worth noting that this section is dedicated solely to reporting these results, with comprehensive analysis and discussion reserved for the following section. On the quantitative front, we offer data-driven insights into the system’s efficiency, latency, and bandwidth requirements. The qualitative results will feature user experiences based on subjective factors, accompanied by visual evidence. In addition, we provide figures related to the quality of crucial steps in the processing pipeline, such as calibration, isolation, and segmentation. These metrics set the stage for a detailed analysis in the subsequent section, aimed at offering a complete assessment of both the technological and practical aspects of our platform in an ever-evolving technological landscape.

### 5.1. Quantitative Results

Initially, we want to provide a notion of the amount of data streaming through the system. As mentioned in Section 4.2.1, each point captured by the Kinect V2 sensors is represented by 15 bytes. Therefore, the volume of a single frame can be calculated as 217,088 × 15 bytes = 3,256,320 bytes = 26,050,560 bits ≈ 26 Mb. In addition, the header volume, as described in Section 4.2.2, must be factored in. This volume is susceptible to variations due to its text-based nature; however, for the frames flowing through the DAN, it is relatively constant and has a mean value of 314 bytes. Therefore, the cumulative transport layer payload per frame equates to 314 + 3,256,320 = 3,256,634 bytes. Our experiments were conducted on a 1 Gigabit data acquisition network, limiting the maximum stable frame rate to eight FPS.

Since TCP was chosen as the transport layer protocol in the DAN, we are obliged to evaluate the effects of this choice. TCP provides a connection-oriented, reliable, stream-based delivery of data and, as such, employs mechanisms for retransmission and network congestion avoidance. These functionalities, although beneficial, can introduce overhead and latency, particularly in the very early stages of the data’s life within the HTPS. To examine these challenges, we conducted a series of experiments to measure the deviation between the sensor’s native frame period (the reciprocal of the frame rate) and the period at which frames are ready for consumption, regardless of the actual rate at which the Processing Pipeline may consume them. In an ideal scenario, these rates would be identical; however, due to factors like retransmission, they can diverge, resulting in a period deviation. These specific metrics are tabulated in Table 4.

**Figure 7:**
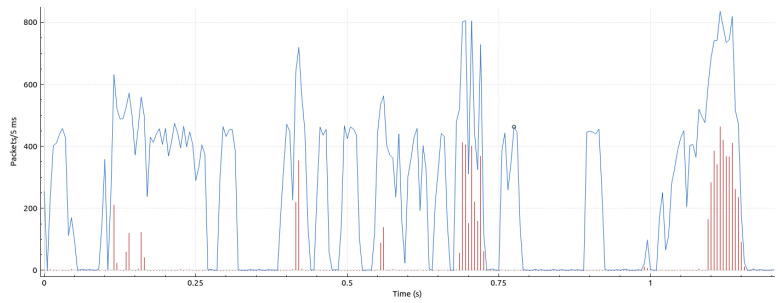
Number of received packets (blue line) and retransmissions (red columns).

**Figure 8:**
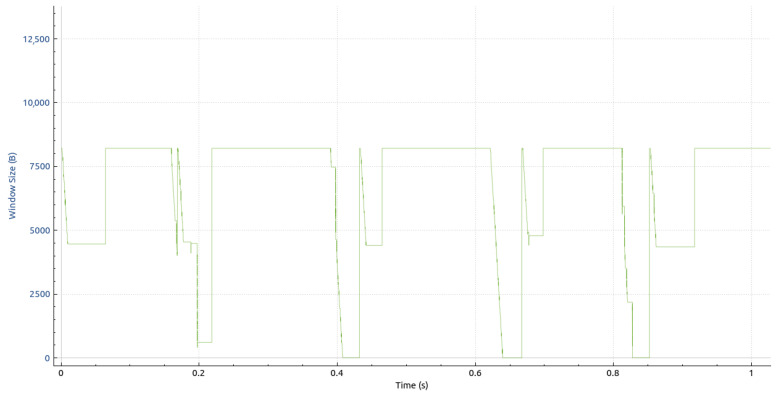
Size of the congestion window with one Sensor Unit in the DAN.

**Figure 9:**
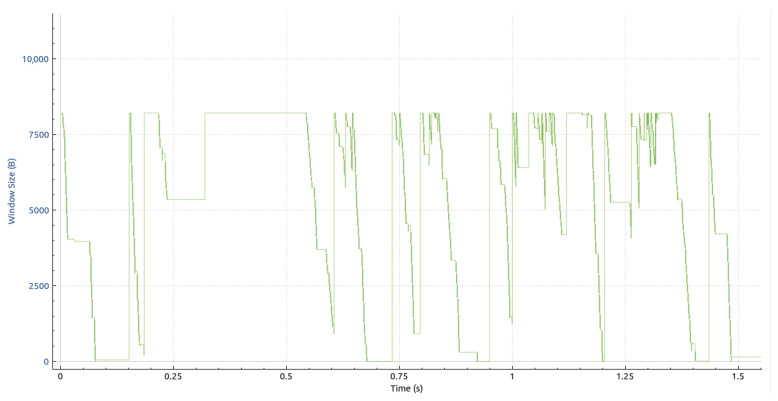
Size of the congestion window with three Sensor Units in the DAN.

In a separate set of experiments, we focused on evaluating the DAN for congestion. We captured key metrics, including the number of packets received by the HPCU, the frequency of retransmissions, and the size of the TCP congestion window. These results are presented in Figure 7, Figure 8 and Figure 9.

The computational steps within the Processing Pipeline introduce significant latency into the data flow, largely due to their computationally intensive tasks and the high data volume they handle. Both the calibration and isolation algorithms are structured into distinct phases, for which we have quantified the computational time. These measurements are detailed in Table 4. Furthermore, we also captured the computational time required by the segmentation process. It is important to highlight that these algorithms have been optimized for GPU execution, with the majority of the computational load being handled by the NVIDIA GeForce RTX 2080Ti GPU.

### 5.2. Qualitative Results

**Figure 10:**
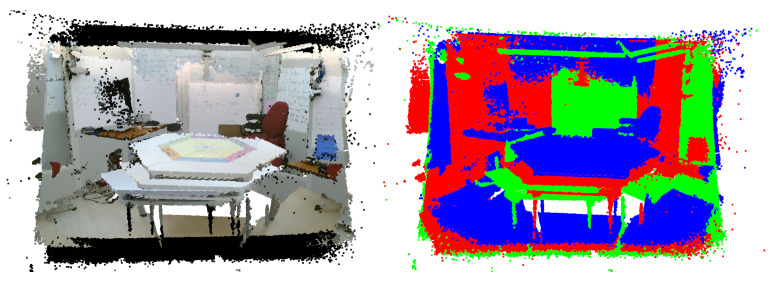
Overlaid PCs representing distinct perspectives of the PME.

The objective of this subsection is to present the qualitative outcomes derived from the practical implementation of our system. As previously noted, the three Kinect V2 sensors that we employ in our setup capture the PME from three distinct perspectives. Consequently, the HPCU obtains three different PCs representing the three different views of the PME overlaid on top of each other, as shown in Figure 10.

**Figure 11:**
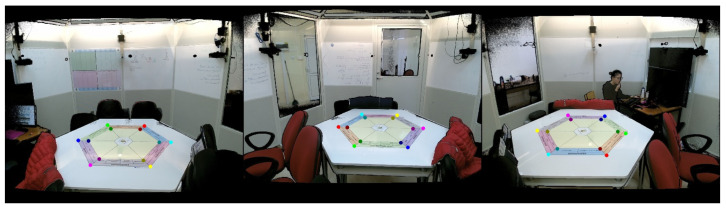
Detected feature points of the object with semantic meaning among different PME perspectives.

To achieve a coherent reconstruction of the environment, these perspectives undergo a calibration process. Specifically, during the offline phase of calibration, we extract RGB images from the PC data of each unique perspective. Each of these images features an object of semantic significance: a table adorned with a printed marker. Subsequently, these images are subjected to analysis by the pre-trained Polygon-YOLOv5 network, which identifies feature points of the semantic object. The network produces 12 feature points per image, enabling point-to-point correspondence across distinct perspectives, as evident in Figure 11, where each corresponding triplet is distinctly marked with a specific color. By leveraging the 2D coordinates and the respective PC data of these feature points, we extract their corresponding positions in the 3D space. They are then employed to calculate the transformation matrix between the distinct perspectives. However, a particular challenge we encountered during the feature detection of the semantic object involved mitigating interference between the infrared (IR) signals emitted by the sensors and strengthened by the object’s high reflectivity. Through experimentation, we identified optimal illumination conditions that facilitated the successful detection of the feature points.

**Figure 12:**
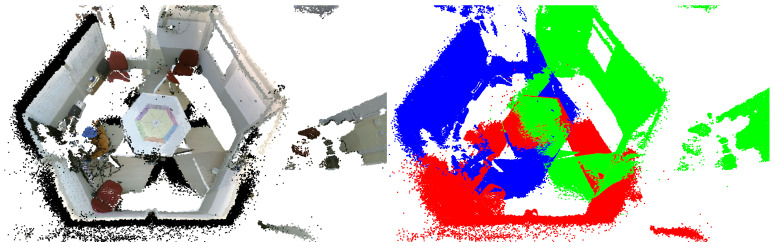
Reconstructed scene after the execution of the calibration step.

The offline calibration phase is a one-time procedure. Nonetheless, it necessitates repetition each time a sensor position changes, as an actual transformation matrix is required. In the online calibration phase, the previously computed transformation is applied continuously to the incoming frames in real-time, facilitated by the GPU in the HPCU. An immediate reconstruction of the PME is generated whenever a set of three PME perspectives enters the online calibration phase, as depicted in Figure 12.

Further, our processing pipeline includes supplementary processing steps, such as scene isolation and object segmentation. While they might not be necessary in other scenarios, they are essential in our specific case.

**Figure 13:**
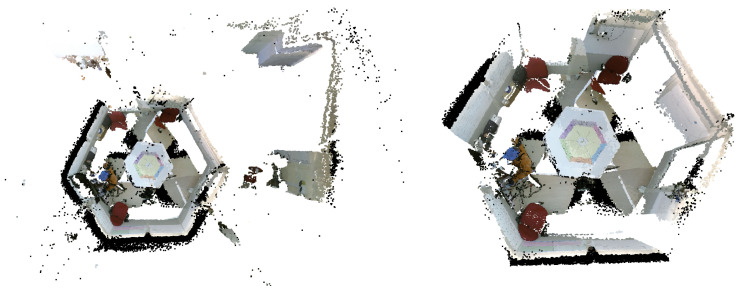
Bee Cube before and after its isolation.

In the process of scene isolation, we eliminate the ambient surroundings encompassing the Bee Cube. The initial computation of the scene extraction parameters is again a one-time, offline procedure. Subsequently, these parameters are persistently applied to the continuously incoming reconstructed scenes, originating from the calibration step. This process also harnesses the GPU capabilities of the HPCU to facilitate its real-time implementation. Figure 13 illustrates the Bee Cube before and after its isolation from its surroundings. This step culminates in the creation of the VME, which is intended to host various virtual objects originating from remote locations in the future.

**Figure 14:**
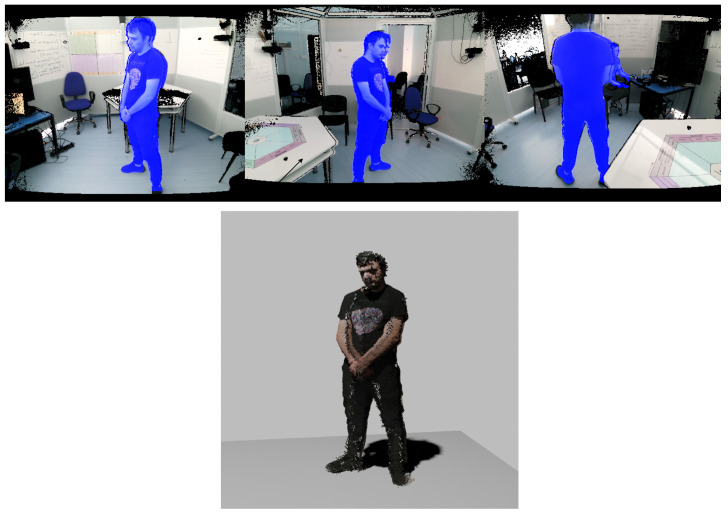
Results from segmentation.

During the segmentation processing step, individuals within the captured PME are separated from the PC data. This process is once more accelerated by the HPCU’s GPU to enable the real-time segmentation. Figure 14 showcases the qualitative results from this step. It is important to emphasize that this operation primarily serves future objectives, where multiple participants from diverse locations will be integrated into the purpose-designed VME.

**Figure 15:**
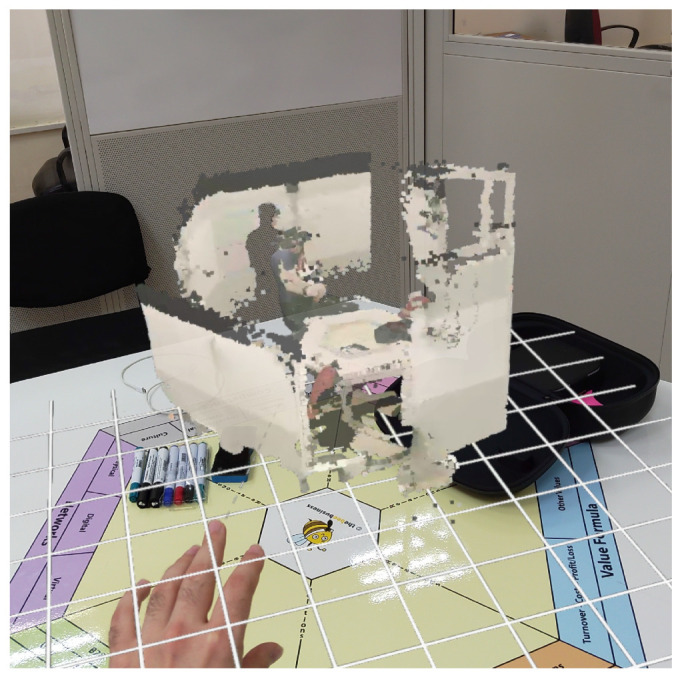
The VME observed in AR.

Finally, Figure 15 provides a visual representation of the VME as observed from various viewpoints using the Hololens 2.

## 6. Discussion

The size of the data frames poses a substantial challenge to the system’s performance. To enhance the frame rate, it would be beneficial to either incorporate a compression algorithm in the Data Pre-processing block of the Sensor Unit or adopt a high-bandwidth DAN. Despite this limitation, our custom application layer protocol has minimal overhead while successfully offering a flexible framework for holographic metadata, underscoring its utility throughout the HTPS architecture.

Regarding the choice of TCP for data transmission, while it assures reliable delivery, it also exacerbates network congestion. This leads to stuttering and desynchronization between the Sensor Units. As evidenced by a period deviation of 27 ms against a frame period of 125 ms—accounting for a 21% deviation in perceived frame rate—the current approach is suboptimal. Moreover, Figure 7 demonstrates that the packet arrival pattern lacks the expected periodicity, indicating non-uniform data delivery.

The congestion window size, as shown in Figure 8, further elucidates this issue. Although congestion is minimized when only one Sensor Unit is active, incorporating additional units causes the window size to fluctuate unpredictably. This is largely attributable to the Sensor Units’ inability to coordinate their transmissions, leading to a TCP Incast scenario where the Data Collector’s buffer overflows. A more efficient transport layer protocol is, therefore, needed to mitigate these issues.

On the computational side, the online phases for calibration, isolation, and segmentation consume significant processing time. This is due, in part, to the use of Python, an interpreted language, for the implementation of these processes. Nonetheless, the visual quality of the processed scenes remains commendable, as evidenced in Figure 10, Figure 11, Figure 12, Figure 13, Figure 14 and Figure 15.

Finally, the architectural design of the HTPS demonstrates a proof of concept that embraces modularity. While modularity introduces some inefficiencies, such as restrictive structures, it also provides explicit definitions for ABs that guide the functionality of the system. This ensures that the HTPS meets predefined requirements while allowing flexibility in both software and hardware implementation. Future work should continue to adopt this modular approach to build upon these foundational insights.

## 7. Conclusions

In this paper, we have embarked on an extensive exploration of the field of HTC. We began by surveying the current landscape of technologies that form the base of HTC, including but not limited to VR, AR, AI, and the emerging 6G networks. The lack of a standardized architecture and modular systems in HTC led us to propose a versatile, modular, and interoperable system architecture designed to address these challenges effectively. Our work delineates the essential ABs for an HTPS and presents a practical implementation of this architecture in a fully functional HTPS platform. Our research took a critical look at both the quantitative and qualitative aspects of our platform’s performance. While the system demonstrates promising results, we also identify critical challenges such as network congestion, frame rate bottlenecks, and processing inefficiencies. The discussion section articulates potential solutions to these challenges, advocating for a more efficient transport layer and optimized data processing workflows.

In alignment with the goals of the HOLOTWIN Project, our platform serves as a catalyst for encouraging research and innovation in the field of HTC. By synthesizing our observations and insights, we reassert our central thesis: The path to realizing the full potential of HTC lies in embracing interoperability through modularity. This approach not only enables streamlined development but also promotes a collaborative ecosystem for research and development, thereby catalyzing the market entry of commercially viable HTC products.

## Figures and Tables

**Table 1:** HTC deployment scenarios.

Scenario	Low-Interactive HTC	High-Interactive HTC	HTC Broadcasting
Description	While it permits higher latency, this approach finds its niche where real-time engagement is not paramount, such as in virtual museum tours. Infrequent content updates and non-urgent data transfer form its backbone.	Necessitating near-instantaneous data transmission, this scenario facilitates environments like remote robotic control, where swift and reliable interaction is indispensable. The demarcation from its low-interactive counterpart hinges on the strict latency and real-time communication prerequisites.	Despite a commonality with low-interactive HTC in latency lenience, this format diverges by providing for larger audiences without necessitating user interaction, as seen in scenarios like public event broadcasts.
Applications	Remote Education Virtual Museums Remote Presentations	Telemedicine Remote Control of Robots Real-Time Holographic Collaboration	Live Sports Matches Public Ceremonies Large Scale Educational Broadcasts
Requirements	Tolerant to longer latency (up to 1 s) Moderate to high bandwidth (several megabits to gigabits per second) Single user to small group experience	Ultra-low latency (sub-millisecond to a few milliseconds) Extremely high bandwidth (gigabits to terabits per second) One-to-one or small group interaction	Tolerant to longer latency (several seconds to a minute) High bandwidth (gigabits to terabits per second) Large audience size (thousands to millions of viewers)

**Table 2:** Architectural blocks proposed in [22].

Architectural Block	Description	Hardware Components	Software Tools/Frameworks
Radiometric Sensor	Capturing both depth and color information of a given scene. Its role is pivotal in realizing the environment and it serves as the primary input to the system.	Microsoft Kinect 2	Microsoft Kinect SDK–Sensor Driver
Sensor Controller	Processing the raw input from the radiometric sensor and managing data handoff to the system. Beyond mere data transfer, it is also responsible for processing and for local connectivity functions.	Intel NUC, Core i7 processor, 8 GB RAM	Finite-state machines
Local Connectivity	Facilitating efficient data communication, ensuring seamless transmission from the sensor controllers to the system’s central computational units. Its role, while not overtly computational, remains essential to maintaining data integrity and timely transfers.	Gigabit Ethernet	MQTT, KCP protocol
Local Controller	A hub for the sensor data streams, emphasis on more complex functions like processing and data fusion. It aggregates information from all the sensors, potentially fusing it before dispatching it to the next stages.	High-performance computing unit, AMD RYZEN 2990WX 32-core processor, 64 GB DDR4 RAM, NVIDIA GeForce RTX 2080Ti DUKE GPU	Hierarchical state machine
Mixed-Reality Server	Synthesizing local and remote scene data to generate a singular, cohesive mixed-reality feed. It not only orchestrates the entire process but also ensures that the generated content is primed for the end-users.		Python, C++
Operator Dashboard Server	A control tower for the entire system. While it does not delve into the intricacies of processing, it provides the necessary oversight, monitoring, and control over the MRTS.		FastAPI, React, InfluxDB
Global Connectivity	Manages data transfer between the local mixed-reality server and remote systems. Ensures secure, synchronized, and timely data exchange for real-time remote collaborations and interactions within the mixed-reality environment.	Internet with Ethernet connection	WebRTC
MR Display	The user’s window into the mixed-reality world. Its function is purely output-driven, translating the processed information into a format that is perceivable and interactive for the user.	Microsoft’s Hololens 2 HMD	Unity

**Table 3:** Explicit responsibilities of architecture blocks.

Architecture Block	Explicit Responsibility
Sensors	Capture designated types of physical characteristics (e.g., audio, visual, depth) from the PME without any interpretation or modification.
Sensor Drivers	Provide a programming interface for control, management, and data services for specific sensor hardware.
Data Pre-processing	Provide a scalable, abstract framework that enables easy integration of a variety of processing algorithms.
Data Streamer	Transmit pre-processed sensor data to the Data Collector over the DAN ensuring data integrity and timely arrival.
Sensor Controller	Coordinate real-time operations among multiple sensors within a unit, and execute commands from the Management Plane without manual intervention.
Data Collector	Aggregate incoming data streams from all sensor units, serving as a singular gateway to pass data onto the Processing layer.
Synchronization	Coordinate Data Streamers to ensure that samples from different sensor units arriving at the Data Collector are temporally aligned within a specified threshold.
Processing Pipeline	Provide a scalable, abstract framework that enables the integration of a variety of processing algorithms, irrespective of whether the execution is local or cloud-based. The framework should be designed to unify both local and cloud-based processing in such a way that the location of execution is transparent to the integration process.
Cache	Provide temporary storage for data (sensor samples and scenes) for fast and efficient retrieval during processing.
Data Streamer	Utilize the Transmission Functions framework to contribute participant-specific, processed data to the unified VME.
Communication Interface	Provide an abstract and transparent interface to serve as the gateway into the Transmission Functions framework, allowing connectivity for all participating sites.
Session Control	Manage the initialization, maintenance, and termination of virtual sessions, including the verification of participant authorizations and ensuring uninterrupted connectivity for all session participants.
Synchronous Scene Fusion	Aggregate and synchronize individual virtual scenes from each participant into a unified, coherent scene, combining both the VME and participant avatars.
Incremental Dispatcher	Detect and dispatch only incremental updates to the VME and avatar characteristics.
Interactive Environment Manager	Aggregate and synchronize Interactive Interfaces from all participating sites to create a coherent global interactive environment.
Storage	Repository for temporarily storing holographic content, session metadata, and user interactions, ensuring high-speed data retrieval throughout the session.
Data Receiver	Utilize the Transmission Functions framework to receive the synchronized, collective VME and participant avatars.
Decompression	Decompress incoming data streams in real-time.
Scene Construction and Alignment	Deinterleave and reinterpret incoming data streams to reconstruct the scene. Align the scene with the participant’s PME. Prepare for delivery to appropriate sensory interfaces.
Scene Rendering	Convert the geometric representations of the scene into visual formats in real-time.
Head-Mounted Displays	Serve as the physical interface for the user, enabling real-time, 6DoF display and interaction within the MR environment, adaptable to AR or VR use cases.
Interactive Interface	Provide a local interactive user interface within the VME that is constantly synchronized with the global interactive environment, managed by the Interactive Environment Manager.
Control Message Server	Distribute control messages and commands to sensor units via the DAN.
Local Controller	Determine and execute the appropriate control actions based on inputs from the Management Interface, the current states of sensor units, the state of the processing pipeline, the state of the data channel, etc.
Transmission Management	Manage and remotely configure the Transmission Functions framework.
MR Interactive Management	Allow participants to manage system settings within the VME without disrupting the immersive experience.
Evaluation Viewport	Enable real-time system evaluation and observation for administrators, allowing them to monitor the impacts of changes in system parameters.
Management Interface	Provide a comprehensive control interface for administrators, enabling control and customization across all layers of the HTPS.

**Table 4:** Quantitative results.

Measurement	Value
Data Collector, Sensor FPS = 8, Period deviation (ms)	27
Calibration—Offline Phase, Computational time (s)	161
Calibration—Online Phase, Computational time (ms)	126
Isolation—Offline Phase, Computational time (s)	3419
Isolation—Online Phase, Computational time (ms)	140
Segmentation, Computational time (ms)	30

Note: All values presented in Table 4 are derived by averaging a total of 50 measurements for each corresponding metric.

## Data Availability

Not applicable.

## Abbreviations

The following abbreviations are used in this manuscript:

3D	Three-dimensional
AB	Architectural block
AI	Artificial intelligence
API	Application programming interface
AR	Augmented reality
AU	Aarhus University
CIT	Classical information theory
CPU	Central processing unit
CV	Computer vision
DAN	Data acquisition network
DASH	Dynamic adaptive streaming over HTTP
DLL	Dynamic-link library
E2E	End-to-end
FPS	Frames per second
GPU	Graphical processing unit
HAS	HTTP adaptive streaming
HDRP	High Definition Render Pipeline
HMD	Head-mounted display
HPCU	High-performance computing unit
HTC	Holographic-type communication
HTPS	Holographic telepresence systems
IR	Infrared
JSON	JavaScript Object Notation
LAN	Local area network
MEC	Mobile edge computing
MPEG	Moving Picture Experts Group
MR	Mixed reality
MRTK	Mixed-reality toolkit
MVP	Minimal viable products
NIR	Near infra-red
NeRF	Neural radiance fields
PC	Personal computer
PCA	Principal component analysis
PME	Physical meeting environment
QoE	Quality of experience
SAND	Server and network-assisted DASH
SDL	Software development kit
SDN	Software-defined networking
TCP	Transmission Control Protocol
UI	User interface
UNiS	University of Surrey
V-PCC	Video-based point cloud compression
VME	Virtual meeting environment
VR	Virtual reality
